# Risk Factors of Going Missing in People Living With Dementia: A Scoping Review

**DOI:** 10.1093/geroni/igab046.2432

**Published:** 2021-12-17

**Authors:** Noelannah Neubauer, Hector Perez, Antonio Miguel-Cruz, Christine Daum, Samantha Dawn Marshall, Elyse Letts, Lili Liu

**Affiliations:** 1 University of Waterloo, Waterloo, Ontario, Canada; 2 University of Alberta, Edmonton, Alberta, Canada; 3 University of Waterloo, Edmonton, Alberta, Canada; 4 University of Waterloo, University of Waterloo, Ontario, Canada

## Abstract

Critical wandering is common in persons living with dementia, it is defined as wandering that results in an individual going missing. This exposes the missing vulnerable older adult to risks and dangers. Persons with dementia who become lost and go missing and get lost can face adverse outcomes, such as injury and death, yet the amount of information available on the risk factors associated with these incidents is scarce. The aim of this study was to identify the risk factors associated with critical wandering in persons living with dementia. We used Tricco et al.’s (2018) approach for scoping reviews and searched the following databases: Medline, EMBASE, CINAHL, and Scopus. We included studies that referred to critical wandering in persons with dementia, cognitive impairment, or Alzheimer, and published since 1980. We identified 3,376 publications, which was reduced to 1641 publications after we removed duplications. A total of 78 studies met the inclusion and exclusion criteria for analysis and extraction. A rigorous process to synthesize and categorize the research evidence was followed. We identified four different types of risk factors associated with going missing: (1) personal, (2) physical environment and geographical location, (3) cultural environment, and (4) social environment and support resources. Recognition of these risk factors can help persons living with dementia and their care partners identify interventions and proactive strategies to mitigate or prevent critical wandering. This will support persons with dementia, their care partners, and community organizations to balance safety, autonomy, and independence to maximize quality of life.

